# Guide to Enhanced Recovery for Cancer Patients Undergoing Surgery: ERAS and Oesophagectomy

**DOI:** 10.1245/s10434-021-10384-5

**Published:** 2021-10-19

**Authors:** Krishna Moorthy, Laura Halliday

**Affiliations:** Department of Surgery and Cancer, Imperial College London, London, UK

## Abstract

Enhanced Recovery After Surgery (ERAS) protocols are widely used in oesophageal cancer surgery. Multiple studies have demonstrated that ERAS protocols are associated with a shorter length of stay and a reduction in the incidence of post-operative complications after oesophagectomy. However, there is substantial heterogeneity in the content of ERAS protocols and the delivery of these pathways can be challenging. This paper discusses the key recommendations for ERAS protocols in oesophageal cancer surgery and the barriers and facilitating factors for their successful implementation.

Oesophageal cancer surgery involves prolonged, complex operations. Patients are often elderly and have multiple co-morbidities.^1–3^ They frequently require neoadjuvant chemotherapy, which further impairs cardiorespiratory fitness.^4,5^ All of these factors contribute to a challenging recovery from surgery. Up to 60% of patients experience post-operative complications,^1,6^ and there can be a substantial decline in functional capacity and quality of life after surgery.^7–9^

Consequently, there is clear need for interventions to improve peri-operative outcomes in these patients. Enhanced Recovery After Surgery (ERAS) protocols are designed to accelerate recovery after surgery. Over the last decade, many studies have examined the impact of ERAS protocols in oesophagectomy patients. There is a large body of evidence showing a significant reduction in length of stay when ERAS protocols are used,^10–14^ with a mean difference of between 1.1 and 3.6 days,^11,12,14^ and no significant difference in readmission rates.^11,12,14^ ERAS pathways are also associated with a significant reduction in the incidence of non-surgical complications,^14^ and respiratory complications in particular.^11,14^ In keeping with the effects on length of stay and complications, there is growing evidence of economic benefits, with per-patient cost reductions of up to US $4000 when ERAS protocols are used.^13,15^

The findings in the literature, however, have been varied, and the magnitude of the effect varies between studies.^11^,^12^ There is substantial heterogeneity in the content of ERAS protocols,^10,11,13,14^ which may explain this variation. Some of the differences may also be due to variable success in the implementation of protocols.^16^ Compliance with ERAS protocols for oesophagectomy patients ranges from 56% to 78%, with up to 20% of patients having a major protocol deviation.^10,15^ Across a range of surgical specialities, higher compliance with ERAS protocols is associated with shorter length of stay after surgery.^17,18^

## Key Participants in Successful ERAS Pathway

ERAS protocols are complex socio-technical interventions that are affected by many different social, organisational and cultural factors. Successful implementation requires engagement with a large number of individuals and specialities,^19^ all of which will make a contribution towards achieving compliance with the protocols. In the context of oesophagectomy pathways, this includes surgeons, anaesthetists, nursing staff, physiotherapists, dieticians, radiologists and pharmacists.

The list of stakeholders will depend on not just the elements in the ERAS protocol but also the various hand-offs between different clinical specialties. For example, in some centres, oesophagectomy patients may go to an intensive care or high-dependency unit for the immediate post-operative recovery period, while in others they may go directly to a surgical post-operative unit. Both patient pathways will involve different individuals, specialties and thus different norms, priorities and practices.

Patients are not passive recipients of care, and patient involvement in ERAS pathways is essential. Compliance with ERAS elements that require patient participation is lower than those that do not require input from patients.^20^ Patient education is an important pre-operative element of ERAS pathways, ensuring that patients know what to expect during their post-operative recovery. However, patient involvement should extend beyond this, from identifying their individual patient-specific barriers for recovery, to patient representation in programme-level pathway design and implementation. Strategies for patient engagement in this process can include focus group interviews and collaborative events with members of the multidisciplinary team (MDT).

## Required Items in ERAS Pathway

There is considerable heterogeneity between the protocols used at different centres,^10,11,13,14^ and there is often no clear definition of each element within the protocol.^12,14^ There are a number of reasons why clear definitions are important. Firstly, they ensure that everyone within the MDT, including the patient, are working towards the same goal. Secondly, from a research perspective, clear definitions are needed to perform robust meta-analysis of study results and ascertain the true impact of each element.^12^ Thirdly, they aid implementation of findings from research studies into routine clinical practice. Finally, poor definitions limit the ability to determine compliance for quality improvement purposes.^21^

The presence of the following elements within the ERAS protocol is significantly associated with a reduction in length of stay after oesophagectomy: immediate extubation, mobilisation ≤ 1 day after surgery, removal of urinary catheter ≤ 2 days, commencing oral intake of fluid ≤ 1 day, enteral supplemental feeding ≤ 1 day, removal of epidural ≤ 4 days, and performing a contrast swallow ≤ 5 days.^12^

In 2018, the ERAS^®^ Society evaluated the evidence for 39 pathway components for oesophagectomy patients.^22^ The recommendations include ERAS concepts that are common across many surgical specialities, such as patient counselling, advice on smoking and alcohol cessation, avoiding prolonged pre-operative fasting and intra-operative hypothermia, and continuous audit of processes and outcomes. The key recommendations for oesophagectomy specific components are summarised in Table 1.

**Table 1:** Oesophagectomy-specific ERAS recommendations

ERAS component	Recommendation
*Pre-admission*	
Nutrition	Assessment and treatment based on individual risk; routine use of immunonutrition is not recommended
Haemoglobin optimisation	Oral iron supplementation for iron-deficiency anaemia
Prehabilitation	Multimodal prehabilitation: exercise programme, personalised nutritional support, psychological support
Timing of surgery	3–6 weeks after neoadjuvant chemotherapy or 6–10 weeks after neoadjuvant radiotherapy
*Pre-operative*	
Bowel preparation	Not to be used routinely
Fasting	Solid food allowed until 6 h prior to surgery (caution if any dysphagia); clear fluids until 2 h prior to surgery
*Intra-operative*	
Minimally invasive surgery	Recommended where there is appropriate training and expertise
Oesophageal reconstruction	Gastric conduit as first-line option
Lymphadenectomy	Two-field lymphadenectomy for T1b–T4 adenocarcinoma in the middle or lower third of the oesophagus
Conduit decompression	Nasogastric tube decompression is recommended
Chest drain placement	Single drain as effective as two and produces less discomfort
Intravenous fluid replacement	Balanced fluid replacement strategies using crystalloid fluids
Muscle relaxants	Intermediate acting neuromuscular blockers
Lung-protective ventilation	Low tidal volumes (6–8 ml/kg) and 2–5 cmH_2_O PEEP
Temperature	Maintain core temperature > 36 °C
*Post-operative*	
ICU/HDU	Level of care should be personalised according to individual patient risk factors
Analgesia	Thoracic epidural as first line; paravertebral blocks are an alternative
Nutrition	Early enteral feeding; aim to achieve full calorie requirements by day 3–6; either jejunostomy or nasojejunal tube may be used
Mobilisation	Early mobilisation with defined daily incremental increases in activity; start on day of surgery if feasible
Removal of chest drains	Remove once draining < 200 ml/day and no evidence of air or chyle leak
Fluid management	Avoid positive fluid balance
Glycaemic control	Target blood glucose < 10 mmol/l
VTE prophylaxis	Continue for 4 weeks after surgery

*PEEP* positive end-expiratory pressure, *ICU* intensive care unit, *HDU* high-dependency unit, *VTE* venous thromboembolism

Many technical and procedural aspects of oesophageal cancer surgery differ between surgical centres.^23–25^ However, guidelines have emerged for many operative considerations. For example, two-field lymphadenectomy is recommended for adenocarcinoma of the middle or lower oesophagus, and a gastric tube is recommended as the first-line choice of conduit for oesophageal reconstruction.^22,25^ The ERAS^®^ Society guidelines highlight that open, minimally invasive and hybrid approaches to oesophagectomy all have acceptable outcomes.^22^ Growing evidence from several systematic reviews and meta-analyses suggests that minimally invasive surgery is associated with fewer post-operative pulmonary complications, reduced blood loss and shorter length of stay, with no difference in the rate of anastomotic leak and comparable lymph node yields.^22,25–28^ The use of minimally invasive surgery has been recommended where there is appropriate training and expertise.^10^

Recommendations for anaesthetic management include the use of intermediate acting neuromuscular blockades, crystalloid fluid replacement, balanced fluid replacement strategies and lung-protective strategies, such as low tidal volumes (6–8 ml/kg) and 2–5 cmH_2_O positive end-expiratory pressure (PEEP).^10,22^ Both inhaled and intravenous anaesthesia are effective for anaesthesia maintenance.^22^

There is currently insufficient evidence to provide recommendations on routine pre-operative cardiopulmonary exercise testing, inspiratory muscle training, intra-operative pyloroplasty or to advocate a specific route for post-operative enteral feeding.^10,22^ In this rapidly growing area of peri-operative research, it is important that protocols are regularly reviewed and adapted in light of the most recent available evidence. Over time, new concepts may be added, and previous elements removed or amended.

## Barriers to and Facilitators of ERAS

The implementation of an ERAS protocol for oesophagectomy patients is challenging.^29^ Multiple barriers to delivering ERAS protocols have been identified, including staff and patient education, MDT communication, resistance to change amongst staff and lack of resources.^30^ In oesophagectomy patients, these difficulties may be compounded by the complexity of the surgery, patient frailty, the large number of interventions performed in the peri-operative period and the large number of clinical staff who are involved in delivering care. To date, there has been very little research to examine strategies to overcome these problems. We propose that recommendations for successful implementation include adaption to the local context, staff engagement, patient engagement and continuous data measurement and feedback.^29^

Continuous monitoring and reporting of compliance is a crucial first step to improve ERAS implementation. Successful implementation should be driven by a methodical approach grounded in the principles of continuous quality improvement. This should be a data-driven process, assessing the population-level compliance and clinical outcomes in parallel to identify areas of strength and areas for improvement and allow assessment of the effectiveness of changes that are made to the pathway’s implementation strategy. Real-time compliance data for each patient can also be utilised within an MDT structure to identify patients who are not meeting their ERAS goals and allow early intervention to improve compliance and recovery.

Stakeholder engagement is essential to the implementation of ERAS protocols, but the engagement needs to also extend to their development. Rather than simply applying an intervention that has worked elsewhere, a system of ‘facilitated evolution’ is advocated to translate and adjust solutions to the local setting.^31,32^ Iterative changes can be made using plan–do–study–act or knowledge-to-action cycles, which are well-recognised techniques to support translating evidence into routine clinical practice.^33,34^ Obtaining input and perspective from a broad spectrum of stakeholders will provide a more comprehensive understanding of the site-specific barriers to ERAS and may help to generate a wider range of ideas on how to overcome them.

Specialist centres can act as clinical silos, with little exchange of ideas and experiences between teams despite having common challenges and goals. Local and regional collaborative forums should be encouraged to share experiences of ERAS implementation for oesophagectomy patients. Through collaborative learning, surgical centres can identify common barriers and develop interventions to overcome them, whilst also obtaining different and fresh perspectives on individual site-specific barriers.

Finally, the peri-operative pathway should be viewed as a continuum. The growing field of prehabilitation is changing how we view pre-operative ERAS components. Prehabilitation uses time between diagnosis and surgery to optimise a patient’s functional capacity, improve psychological wellbeing and foster greater patient engagement in their treatment.^25,35^ With growing evidence of benefit from prehabilitation in oesophageal cancer patients,^25,36^ greater integration of prehabilitation and ERAS pathways will help to embed the notion of proactive, goal-directed, patient-centred care throughout the patient’s treatment and recovery.

## Summary

- ERAS pathways are associated with shorter length of stay and reduced incidence of respiratory complications following oesophagectomy;
- Clear definitions are needed for each element in the ERAS protocol;
- Immediate extubation, mobilisation ≤ 1 day after surgery, removal of urinary catheter ≤ 2 days, commencing oral intake of fluid ≤ 1 day, enteral supplemental feeding ≤ 1 day, removal of epidural ≤ 4 days and performing a contrast swallow ≤ 5 days are all associated with reduced length of stay;
- Many barriers to ERAS implementation will be site-specific factors, thus the implementation strategy must be tailored to the local context;
- Multidisciplinary team engagement in both ERAS pathway design and the day-to-day delivery of ERAS are key to successful implementation;
- Real-time monitoring of compliance should be used to provide a continuous audit of implementation;
- ERAS^®^ Society guidelines for perioperative care in esophagectomy are available from https://doi.org/10.1007/s00268-018-4786-4.

## References

[1] Low DE, Kuppusamy MK, Alderson D (2019). Benchmarking complications associated with esophagectomy. Ann Surg.

[2] Backemar L, Lagergren P, Johar A, Lagergren J (2015). Impact of co- morbidity on mortality after oesophageal cancer surgery. Br J Surg.

[3] McIsaac DI, Bryson GL, van Walraven C (2016). Association of Frailty and 1- Year Postoperative mortality following major elective noncardiac surgery: a population-based cohort study. JAMA Surg.

[4] Sinclair R, Navidi M, Griffin SM, Sumpter K (2016). The impact of neoadjuvant chemotherapy on cardiopulmonary physical fitness in gastro-oesophageal adenocarcinoma. Ann R Coll Surg Engl.

[5] Hoppe S, Rainfray M, Fonck M (2013). Functional decline in older patients with cancer receiving first- line chemotherapy. J Clin Oncol.

[6] Sinclair RCF, Phillips AW, Navidi M, Griffin SM, Snowden CP (2017). Pre-operative variables including fitness associated with complications after oesophagectomy. Anaesthesia.

[7] Barbour AP, Lagergren P, Hughes R, Alderson D, Barham CP, Blazeby JM (2008). Health-related quality of life among patients with adenocarcinoma of the gastro- oesophageal junction treated by gastrectomy or oesophagectomy. Br J Surg.

[8] Akkerman RDL, Haverkamp L, van Rossum PSN, van Hillegersberg R, Ruurda JP (2015). Long- term quality of life after oesophagectomy with gastric conduit interposition for cancer. Eur J Cancer.

[9] Minnella EM, Awasthi R, Loiselle S, Agnihotram RV, Ferri LE, Carli F (2018). Effect of exercise and nutrition prehabilitation on functional capacity in esophagogastric cancer surgery: a randomized clinical trial. JAMA Surg.

[10] Findlay JM, Gillies RS, Millo J, Sgromo B, Marshall REK, Maynard ND (2014). Enhanced recovery for esophagectomy: a systematic review and evidence- based guidelines. Ann Surg.

[11] Markar SR, Karthikesalingam A, Low DE (2015). Enhanced recovery pathways lead to an improvement in postoperative outcomes following esophagectomy: systematic review and pooled analysis. Dis Esophagus.

[12] Markar SR, Naik R, Malietzis G, Halliday L, Athanasiou T, Moorthy K (2017). Component analysis of enhanced recovery pathways for esophagectomy. Dis Esophagus.

[13] Gemmill EH, Humes DJ, Catton JA (2015). Systematic review of enhanced recovery after gastro-oesophageal cancer surgery. Ann R Coll Surg Engl.

[14] Pisarska M, Malczak P, Major P, Wysocki M, Budzynski A, Pedziwiatr M (2017). Enhanced recovery after surgery protocol in oesophageal cancer surgery: systematic review and meta-analysis. PLOS ONE.

[15] Lee L, Li C, Robert N (2013). Economic impact of an enhanced recovery pathway for oesophagectomy. Br J Surg.

[16] Paton F, Chambers D, Wilson P (2014). Effectiveness and implementation of enhanced recovery after surgery programmes: a rapid evidence synthesis. BMJ Open.

[17] Smart NJ, White P, Allison AS, Ockrim JB, Kennedy RH, Francis NK (2012). Deviation and failure of enhanced recovery after surgery following laparoscopic colorectal surgery: early prediction model. Colorectal Dis.

[18] Simpson JC, Moonesinghe SR, Grocott MPW (2015). Enhanced recovery from surgery in the UK: an audit of the enhanced recovery partnership programme 2009–2012. Br J Anaesth.

[19] Gramlich LM, Sheppard CE, Wasylak T (2017). Implementation of enhanced recovery after surgery: a strategy to transform surgical care across a health system. Implement Sci.

[20] Thorn C, White I, Burch J, Malietzis G, Kennedy R, Jenkins J (2016). Active and passive compliance in an enhanced recovery programme. Int J Colorectal Dis.

[21] Hulscher MEJL, Laurant MGH, Grol RPTM (2003). Process evaluation on quality improvement interventions. Qual Saf Health Care..

[22] Low DE, Allum W, De Manzoni G (2019). Guidelines for perioperative care in esophagectomy: Enhanced Recovery After Surgery (ERAS®) Society recommendations. World J Surg..

[23] Halliday LJ, Doran SLF, Sgromo B (2020). Variation in esophageal anastomosis technique-the role of collaborative learning. Dis Esophagus.

[24] Varagunam M, Park MH, Sinha S (2019). National Oesophago-Gastric Cancer Audit 2018.

[25] Zylstra J, Boshier P, Whyte GP, Low DE, Davies AR (2018). Peri-operative patient optimization for oesophageal cancer surgery—from prehabilitation to enhanced recovery. Best Pract Res Clin Gastroenterol.

[26] Guo W, Guo W, Ma X (2016). Combined thoracoscopic-laparoscopic esophagectomy versus open esophagectomy: a meta-analysis of outcomes. Surg Endosc.

[27] Nagpal K, Ahmed K, Vats A (2010). Is minimally invasive surgery beneficial in the management of esophageal cancer? A meta-analysis. Surg Endosc.

[28] Biere SS, van Berge Henegouwen MI, Maas KW (2012). Minimally invasive versus open oesophagectomy for patients with oesophageal cancer: a multicentre, open-label, randomised controlled trial. Lancet.

[29] Halliday LJ, Markar SR, Doran SLF, Moorthy K. Enhanced recovery protocols after oesophagectomy. J Thorac Dis. 2017 10.21037/jtd.2017.07.16 10.21037/jtd.2017.07.16 PMC5538981 28815074

[30] Pearsall EA, Meghji Z, Pitzul KB (2015). A qualitative study to understand the barriers and enablers in implementing an enhanced recovery after surgery program. Ann Surg.

[31] Ovretveit J (2004). A framework for quality improvement translation: understanding the conditionality of interventions. Jt Comm J Qual Patient Saf.

[32] Damschroder LJ, Aron DC, Keith RE, Kirsh SR, Alexander JA, Lowery JC (2009). Fostering implementation of health services research findings into practice: a consolidated framework for advancing implementation science. Implement Sci.

[33] Ovretveit J (2011). Understanding the conditions for improvement: research to discover which context influences affect improvement success. BMJ Qual Saf.

[34] McLeod RS, Aarts M, Chung F (2015). Development of an Enhanced Recovery After Surgery guideline and implementation strategy based on the knowledge-to-action cycle. Ann Surg.

[35] Carli F, Bessissow A, Awasthi R, Liberman S (2020). Prehabilitation: finally utilizing frailty screening data. Eur J Surg Oncol..

[36] Halliday L, Doganay E, Winter-Blyth V, Hanna G, Moorthy K (2020). The impact of prehabilitation on post-operative outcomes in oesophageal cancer surgery: a propensity score matched comparison. J Gastrointest Surg.

